# Psychometric Validation of the Epworth Sleepiness Scale for Children and Adolescents (ESS-CHAD) in Slovenia: Implications for Public Health Research and Practice

**DOI:** 10.2478/sjph-2026-0003

**Published:** 2026-03-01

**Authors:** Ajda Mlakar, Kaja Gril Rogina, Matic Šmigoc, Barbara Gnidovec Stražišar, Andreja Kukec

**Affiliations:** University of Ljubljana, Faculty of Medicine, Vrazov trg 2, 1000 Ljubljana, Slovenia; University of Ljubljana, Faculty of Medicine, Department of Public Health, Zaloška cesta 4, 1000 Ljubljana, Slovenia; National Institute of Public Health, Ljubljanska ulica 4, 2000 Maribor, Slovenia; Javni lekarniški zavod Mariborske lekarne Maribor, Minařikova ulica 6, Maribor, 2000 Maribor, Slovenia; University of Maribor, Faculty of Medicine, Taborska ulica 8, 2000 Maribor, Slovenia; General Hospital Celje, Oblakova ulica 5, 3000 Celje, Slovenia

**Keywords:** Excessive daytime sleepiness, Questionnaires, Validation, Adolescents, Slovenia, čezmerna dnevna zaspanost, vprašalniki, validacija, mladostniki, Slovenija

## Abstract

**Introduction:**

This study aimed to develop a suitable instrument for assessing excessive daytime sleepiness (EDS) in Slovenian children and adolescents by translating the ESS-CHAD and psychometrically validating its Slovenian version (ESS-CHAD-SI).

**Methods:**

The ESS-CHAD was translated and back-translated according to established cross-cultural adaptation guidelines, and content validity was assessed by eleven experts from relevant clinical and research disciplines. A nationwide sample of 3,314 adolescents (≈52% females), with an overall mean age of 15.4±1.7 years, completed the questionnaire. Reliability was evaluated using Cronbach's α and Guttman's λ2, and construct validity was examined using exploratory and confirmatory factor analyses.

**Results:**

All items met the predefined thresholds for content relevance, while clarity indices were acceptable for the majority of items. Factor analyses indicated that a two-factor model provided a better fit to the data than the original unidimensional structure, distinguishing between passive sleepiness and more clinically concerning manifestations of sleepiness. The ESS-CHAD-SI demonstrated adequate internal consistency.

**Conclusions:**

The ESS-CHAD-SI is a reliable, valid, and culturally adapted instrument for assessing excessive daytime sleepiness in Slovenian adolescents. The identified two-factor structure enhances its clinical and public health relevance by enabling differentiation between sleepiness related to modifiable sleep behaviours and potentially pathological somnolence. The scale is suitable for use in school-based screening, clinical practice, and epidemiological research.

## Izvleèek

**Uvod:**

Namen študije je bil zagotoviti ustrezen instrument za ocenjevanje čezmerne dnevne zaspanosti (EDS) pri slovenskih otrocih in mladostnikih s prevodom vprašalnika ESS-CHAD ter psihometrično validacijo njegove slovenske različice (ESS-CHAD-SI).

**Metode:**

Vprašalnik ESS-CHAD je bil preveden in vzvratno preveden v skladu z uveljavljenimi smernicami za kulturno adaptacijo, vsebinsko veljavnost pa je ocenilo enajst strokovnjakov z različnih kliničnih in raziskovalnih področij. V nacionalni raziskavi je sodelovalo 3314 mladostnikov (≈ 52 % deklet), s povprečno starostjo 15,4 ± 1,7 let. Zanesljivost je bila ocenjena s koeficientoma Cronbach α in Guttman λ2, konstruktna veljavnost pa z eksploratorno in konfirmatorno faktorsko analizo.

**Rezultati:**

Vse postavke so dosegle vnaprej določene pragove vsebinske relevantnosti, medtem ko so bili indeksi jasnosti za večino postavk sprejemljivi. Faktorska analiza je pokazala, da se dvofaktorski model bolje prilega podatkom kot izvirna enodimenzionalna struktura, pri čemer loči med pasivno zaspanostjo in klinično bolj zaskrbljujočimi oblikami somnolence. Vprašalnik ESS-CHAD-SI se je izkazal za ustrezno notranje skladnega.

**Zaključki:**

ESS-CHAD-SI je zanesljiv, veljaven in kulturno prilagojen instrument za ocenjevanje čezmerne dnevne zaspanosti pri slovenskih mladostnikih. Ugotovljena dvofaktorska struktura povečuje njegovo klinično in javnozdravstveno uporabnost, saj omogoča razlikovanje med zaspanostjo, povezano s spremenljivimi vedenjskimi vzorci spanja, in potencialno patološko somnolenco. Lestvica je primerna za uporabo v šolskih presejalnih programih, klinični praksi in epidemioloških raziskavah.

## 1 INTRODUCTION

Sleep is one of the fundamental human needs and is defined as a reversible and recurring behavioural state in which an individual is perceptibly detached from the environment and does not respond to external stimuli (1). It has been increasingly recognised as a key determinant of public health (2, 3), as humans spend approximately one-third of their lives sleeping, and evidence indicates that insufficient sleep adversely affects both physical and mental health (4). Excessive daytime sleepiness (EDS) is a key symptom reflecting insufficient, fragmented, or misaligned sleep (5). It is defined as a heightened propensity to fall asleep or experience unintentional sleep episodes during the day, even under circumstances when wakefulness is expected (5).

Adolescents are particularly vulnerable due to physiological circadian phase delays, insufficient sleep duration, and social demands such as early school start times (6, 7). Persistent EDS in adolescence is not only associated with acute cognitive and emotional impairments but also with long-term risks for metabolic, cardiovascular, and psychiatric disorders (8). Despite its high prevalence and impact, EDS often remains underrecognised and undertreated in adolescent populations (9). From a public health perspective, EDS increases the risk of accidents, injuries, and diminished quality of life, making it a critical health indicator in youth populations (9). Epidemiological data indicate that EDS is widespread among adolescents. Studies across different populations report prevalence ranging from 20% to 40%, depending on the instruments used and the cut-off points applied (10).

Several validated questionnaires have been developed to measure EDS in children and adolescents. Among the most frequently applied are the Paediatric Daytime Sleepiness Scale (PDSS) (11) and the Epworth Sleepiness Scale for Children and Adolescents (ESS-CHAD) (12). The latter is designed for screening and assessing sleepiness in the general population rather than for diagnostic purposes in clinical samples (13).

Although validated questionnaires for sleep-disordered breathing and chronotype in adults are available (14,15,16) no standardised, validated Slovenian versions of EDS assessment tools for adolescents exist. Preliminary translations of other sleep-related instruments have been attempted (14,15,16), but systematic cultural adaptation and psychometric validation are missing in Slovenian. To address this gap, we intended to conduct a nationwide study examining EDS and related outcomes, including sleep hygiene, sleep quality, and chronotype, in a representative sample of adolescents. Before implementation, it was necessary to prepare an appropriate methodology.

To provide a suitable instrument for the assessment of EDS in Slovene children and adolescents, our study aimed to translate the ESS-CHAD and to validate its Slovenian version (ESS-CHAD-SI) properly.

## 2 METHODS

### 2.1 Study design and time frame

This study was designed as a questionnaire validation study, carried out from October 2023 to April 2024 as part of a larger research project.

### 2.2 Study population and sampling

The study sample was obtained using a multistage, school-based cluster sampling approach. The target population consisted of 8th-grade elementary school students and students in the 1st and 3rd years of secondary school in Slovenia. In the first stage, primary and secondary schools were randomly selected from all Slovenian statistical regions to ensure nationwide geographic coverage. In the second stage, intact classes within participating schools were included, and all students in the selected classes were invited to participate. Overall, 101 primary and secondary schools were invited to participate in the study, and 3,700 adolescents were invited to participate. The target sample size was determined based on an a priori power analysis conducted for the broader nationwide study, of which the present psychometric validation represents one component.

The adolescents completed the ESS-CHAD-SI in a paper-and-pencil format during regular school hours, with the researcher present throughout the session.

### 2.3 Instrument description

ESS-CHAD is a self-report instrument consisting of eight items that assess sleepiness. Two versions of this instrument are available: a general and a research version. For our study, the general version was used.

Children and adolescents aged 7–17 years evaluate, using a four-point Likert scale (0 = would never doze to 3 = high chance of dozing), how often during the past month they experienced daytime sleepiness while engaging in the listed activities. The maximum score on the scale is 24, with higher scores indicating greater daytime sleepiness over the past month (12). A score above 10 is generally considered indicative of excessive daytime sleepiness (17). In a sample of American adolescents, the scale demonstrated high reliability, with an internal consistency (Cronbach's α) of 0.75 (13). The original questionnaire assumes a unidimensional factor structure (12).

### 2.4 Translation of the instrument

#### 2.4.1 Translation process

The translation process was conducted following the methodological guidelines provided by Peters and Passchier (18), and the adaptation and validation procedures followed the guidelines outlined by Sousa and Rojjanasrirat (19).

The scale was first translated into Slovenian and then back-translated into English by two experts – a medical doctor specialised in public health with expertise in sleep measurement terminology, and a pharmacist without specific exposure to medical jargon in the field of sleep measurement - both of whom are native speakers of Slovenian and have near-native proficiency in English (CERF C2). The forward and back translations were performed independently and in a blinded manner (18, 20). After completing the translation process, both translators contributed to resolving discrepancies and ensuring semantic, idiomatic, and conceptual equivalence before the content validation phase (21).

The final output at this stage was ESS-CHAD-SI.

#### 2.4.2 Pre-testing of the translated instrument

Prior to the validation, the instrument was pre-tested in one class of adolescents using a cognitive interviewing approach (21). Participants were encouraged to comment on the clarity and wording of each item as they completed the scale, enabling identification of potential comprehension issues and ensuring cultural and linguistic appropriateness.

### 2.5 Validation of the instrument

#### 2.5.1 Content validity assessment

Eleven experts from various fields evaluated the studied questionnaire's items based on content validity criteria (22, 23). The expert group was field-diverse, consisting of pairs of psychologists, clinical psychologists, paediatricians, neurologists, sanitary engineers, and a public health expert.

Experts independently evaluated the items of the studied questionnaires using a pre-prepared evaluation form for assessing relevance (1 = not necessary or irrelevant; 2 = useful, but should be significantly modified; 3 = useful, but should be partially modified; 4 = essential) and clarity (1 = the item is unclear; 2 = the item needs significant modification; 3 = the item needs partial modification; 4 = the item is clear). They were also encouraged to comment on content and grammar and suggest improvements for individual items. Relevance and clarity analyses were conducted to assess the fulfilment of the content validity criteria. The relevance of the items was assessed using the content validity ratio, as per Equation 1 (22, 23). The threshold for an acceptable content validity ratio for eleven evaluators is 0.59 and 0.62 for ten evaluators (24).

Equation 1. $$\begin{array}{c} \mathrm{CVR}=\frac{x-\frac{N}{2}}{\frac{N}{2}}\\ \begin{array}{l} \mathrm{CVR}=\mathrm{content}\;\mathrm{validity}\;\mathrm{ratio}\\ x=\mathrm{number}\;\mathrm{of}\;\mathrm{evaluators}\;\mathrm{who}\;\mathrm{rated}\;\mathrm{the}\;\mathrm{item}\;\mathrm{as}\;\mathrm{essential}\\ N=\mathrm{numbe}\;\mathrm{of}\;\mathrm{all}\;\mathrm{evaluators} \end{array} \end{array}$$

The clarity index (CI) was calculated as per Equation 2 (22). According to McGartland Rubie et al. (25), the acceptable proportion of agreement regarding the clarity index of an item should exceed 0.80.

Equation 2. $$\begin{array}{c} \mathrm{CI}=\frac{x}{N}\\ \begin{array}{l} \mathrm{CI}=\mathrm{clarity}\;\mathrm{index}\\ x=\mathrm{number}\;\mathrm{of}\;\mathrm{evaluators}\;\mathrm{who}\;\mathrm{rated}\;\mathrm{the}\;\mathrm{item}\;\mathrm{as}\;\mathrm{essential}\\ N=\mathrm{numbe}\;\mathrm{of}\;\mathrm{all}\;\mathrm{evaluators} \end{array} \end{array}$$

Statistical analysis was performed in Microsoft Excel for this part of the validation.

#### 2.5.2 Construct validity and reliability assessment

Construct validity was tested first by exploratory factor analysis (EFA) (principal axis factoring) with orthogonal Varimax rotation with Kaiser normalisation (26,27,28) and later by confirmatory factor analysis (CFA) with the Weighted Least Squares with Mean and Variance adjustment (WLSMV) method (29, 30), comparing the obtained models with those of an original study (12, 13). For a model to be considered as having an acceptable fit, both the Comparative Fit Index (CFI) and the Tucker-Lewis Index (TLI) should reach values ≥ 0.95 (30). The recommended threshold for the RMSEA is below 0.06 (29), although values up to 0.08 are acceptable under more robust criteria (30). The SRMR index is generally considered acceptable, being ≤ 0.07 (30).

The reliability of the questionnaire and individual subscales was assessed using Cronbach's α and Guttman's λ2 coefficients. Following the psychometric criteria proposed by Nunnally and Bernstein (31) reliability coefficients ≥ 0.70 are adequate for early-phase research, coefficients around 0.80 are suitable for group-level analyses, and coefficients ≥ 0.90–0.95 are recommended when measures are used to make decisions about individuals.

IBM SPSS software, version 27, and IBM SPSS AMOS, version 27, were used in this part of the validation. Normality of the variables was confirmed by visual inspection and the Shapiro-Wilk test. Significance was set at p < 0.05.

### 2.6 Ethical considerations

Study design was approved by the Commission of the Republic of Slovenia for Medical Ethics (0120-282/2023/3).

## 3 RESULTS

### 3.1 Description of the study participant group

Out of 3,700 invitees, 3,395 adolescents agreed to participate and completed the questionnaire (response rate 91.8%).

Among participants, 69 (2.03%) reported having a diagnosed sleep disorder, and an additional 12 (0.35%) did not provide information regarding sleep disorder diagnosis. These 83 individuals were therefore excluded from the psychometric validation process.

The analyses were conducted on a final sample of 3,314 adolescents, with a mean age 15.4±1.7 years. Within this sample, 1,707 (51.5%) were female, and 1,511 (45.6%) were male, while 96 (2.9%) participants chose not to disclose their gender. Regarding school grade, 899 (27.1%) were enrolled in the 8th grade of primary school, 1,305 (39.4%) in the 1st year of secondary school, and 1,110 (33.5%) in the 3rd year of secondary school.

### 3.2 Translation and cultural adaptation results

As a result of translation and cultural adaptation, a first draft of ESS-CHAD-SI was prepared. After cognitive interviewing, which showed that the ESS-CHAD-SI was easy to understand, only one modification was suggested (watching TV or video content alongside TV). A final version of ESS-CHAD-SI is available from the first author.

### 3.3 Content validity assessment results

Table 1 reports relevance and clarity indexes for ESS-CHAD-SI. The items that reached the threshold of agreement for each validity index were all eight, except items 5 and 6. The CI values for these two items were relatively low.

**Table 1.** Content validation indices for the Slovenian translation of the Epworth Sleepiness Scale for Children and Adolescents questionnaire.

**Item**	**CVR**	**CI**
**1**	0.82	0.82
**2**	1.00	0.82
**3**	0.82	0.82
**4**	1.00	0.82
**5**	0.82	0.73
**6**	1.00	0.73
**7**	1.00	0.82
**8**	0.82	0.82

Legend: ESS-CHAD-Epworth Sleepiness Scale for Children and Adolescents; CVR-content validity ratio; CI-clarity index.

Means and standard deviations for each item are additionally provided in Table 2. Items 4 and 5 showed the highest average scores, while Items 6 and 8 demonstrated the lowest. Additionally, Pearson correlation coefficients were all statistically significant and positive.

**Table 2.** Descriptive statistics and Pearson correlation coefficients among the Slovenian translation of the Epworth Sleepiness Scale for Children and Adolescents questionnaire.

**Item**	***M***	***SD***	**r**							
			**1**	**2**	**3**	**4**	**5**	**6**	**7**	**8**
**1**	0.70	0.98	1.00							
**2**	1.20	1.04	0.25	1.00						
**3**	0.74	0.97	0.26	0.22	1.00					
**4**	1.39	1.08	0.19	0.23	0.25	1.00				
**5**	1.36	1.17	0.20	0.30	0.31	0.29	1.00			
**6**	0.21	0.57	0.16	0.16	0.31	0.15	0.15	1.00		
**7**	0.96	1.04	0.20	0.31	0.34	0.26	0.64	0.24	1.00	
**8**	0.14	0.51	0.12	0.10	0.24	0.09	0.07	0.51	0.17	1.00

Legend: *M*-mean; *SD*-standard deviation; r-Pearson correlation coefficients

### 3.4 Construct validity assessment results

The assumption of normality was met. The results of the EFA showed that, according to the Kaiser-Guttman criterion, a two-factor solution was identified for the construct of excessive daytime sleepiness. The two extracted factors together accounted for 37.16% of the construct's total variance (Table 3). Subsequently, Table 4 presents the factor loadings for each item on the extracted factors. After Varimax rotation with Kaiser normalisation, it can be observed that the loadings were concentrated; nearly all items loaded most strongly on the first factor. Exceptions were items 6 and 8, which loaded more heavily on the second factor.

**Table 3.** Results of Exploratory Factor Analysis for the Slovenian translation of the Epworth Sleepiness Scale for Children and Adolescents questionnaire.

	**Initial Eigenvalues**			**Extraction Sums of Squared Loadings**			**Rotated Extraction Sums of Squared Loadings**		
		
**Factor**	**Total**	**% of variance**	**Cumulative variance**	**Total**	**% of variance**	**Cumulative variance**	**Total**	**% of variance**	**Cumulative variance**
**1**	2.73	34.13	34.13	2.15	26.92	26.92	1.82	22.72	22.72
**2**	1.31	16.38	50.51	0.82	10.24	37.16	1.15	14.44	37.16
**3**	0.92	11.51	62.02						
**4**	0.80	9.94	71.96						
**5**	0.75	9.39	81.35						
**6**	0.66	8.21	89.56						
**7**	0.48	6.01	95.57						
**8**	0.35	4.43	100.00						

**Table 4.** Factor loading matrix of the Slovenian translation of the Epworth Sleepiness Scale for Children and Adolescents questionnaire items after rotation.

	**Factor matrix with rotation**	
**Factor**	**1**	**2**
**1**	0.32	0.17
**2**	0.43	0.13
**3**	0.43	0.33
**4**	0.40	0.12
**5**	0.78	−0.01
**6**	0.18	0.73
**7**	0.73	0.13
**8**	0.09	0.66

**Figure 1.**
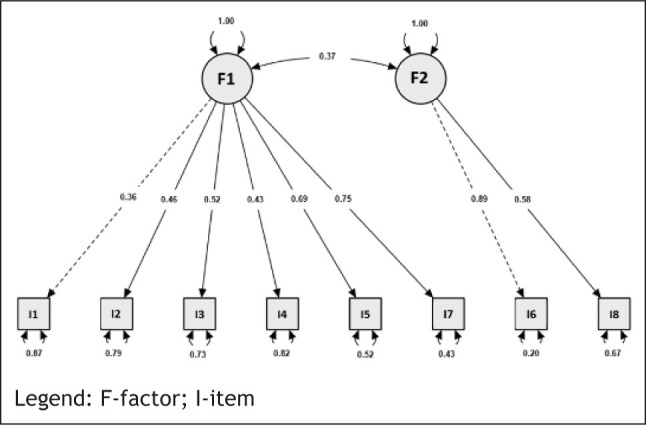
Graphical representation of the two-factor structure of the Slovenian translation of the Epworth Sleepiness Scale for Children and Adolescents questionnaire.

The results of the CFA are presented in Table 5, with graphical representations provided in Figure 1. It is suggested that the one-factor model demonstrated only moderate fit. Although the fit indices approached the recommended thresholds, they did not meet the criteria for good fit. In contrast, the two-factor model, which assumes two latent dimensions, showed superior fit. Both the CFI and TLI indicated satisfactory model fit to the empirical data, and the adequacy of the two-dimensional model was further supported by the SRMR index, which fell below the critical threshold.

**Table 5.** Confirmatory factor analysis indices and descriptive statistics for the Slovenian translation of the Epworth Sleepiness Scale for Children and Adolescents questionnaire.

**Indicator**	**One-factor solution**	**Two-factor solution**
M (SD)	6.65 (4.35)	6.32 (4.00)^*^
		0.34 (0.94)^**^

χ^2^	352.77	262.52
df	20	19
p	< 0.001	< 0.001
CFI	0.94	0.96
TLI	0.92	0.94
RMSEA	0.071	0.063
RMSEA_CI_	0.065–0.078	0.056–0.069
SRMR	0.081	0.046

Legend: CFI-Comparative Fit Index; TLI-Tucker-Lewis Index; RMSEA-Root Mean Square Error of Approximation; SRMR-Standardized Root Mean Squared Residual; RMSEA_CI_-confidence interval of RMSEA;

* Factor 1;

** Factor 2

### 3.5 Reliability assessment results

The results of the reliability (internal consistency) assessment showed that the Cronbach's α coefficient for the ESS-CHAD-SI questionnaire was 0.71, and Guttman's λ2 was 0.73, indicating that the questionnaire is adequate and reliable. Reliability indicators for the individual factors showed acceptable to borderline reliability (α_1_ = 0.71, λ_2,1_ = 0.72); (α_2_ = 0.68, λ_2,2_ = 0.68).

## 4 DISCUSSION

ESS-CHAD-SI proved to be a reliable, valid tool for assessing EDS in adolescents. Its two-factor structure enhances clinical and public health applicability, enabling differentiation between modifiable sleep-hygiene-related sleepiness and potentially pathological somnolence.

In the content validity evaluation of the ESS-CHAD-SI, experts agreed that most of the items were fully appropriate. All items met the relevance threshold, and given that the original questionnaire is unidimensional, it was not meaningful to assess factor validity. Items 5 and 6 demonstrated slightly lower clarity indices, which would most likely have exceeded the threshold had an additional expert been included in the evaluation. The already heterogeneous panel of evaluators could be further strengthened by the inclusion of a psychiatrist and/or child psychiatrist. Despite the generally favourable assessments of the remaining items, experts provided several substantive comments, specifically that items 3, 4, and 8 should be culturally and linguistically adapted. It should also be emphasised that the questionnaire is frequently administered in its original electronic format. Experts highlighted that the use of an electronic questionnaire is less feasible in the Slovenian clinical context. Consequently, the questionnaire was adapted to ensure its applicability in a paper-and-pencil format (12).

When interpreting the results of the factor analysis in our study, we cannot ignore that a two-factor structure emerged, whereas the original questionnaire assumed a unidimensional structure (12). CFA on our dataset indicated that the two-factor model provided a better fit, as its fit indices were closer to the recommended thresholds. Analysis of the collected data revealed that the highest mean scores, indicating the most pronounced daytime sleepiness, occurred for items describing sleepiness while riding in a car or bus and during an afternoon nap. This supports the notion that excessive daytime sleepiness in adolescents is most evident in passive, low-stimulus situations or when adolescents deliberately decide it is time to rest and/or sleep (e.g., during an afternoon nap). This finding aligns with the conceptualisation of sleepiness as a disruption of the balance between homeostatic sleep pressure and the circadian arousal signal (32). In children and adolescents with insufficient nighttime sleep or poor, irregular sleep hygiene, sleepiness first manifests in passive contexts, particularly in the afternoon, when the suprachiasmatic nucleus reduces activation of the orexin–monoamine system that promotes and maintains wakefulness (33).

In contrast, the lowest mean values – and thus the least pronounced daytime sleepiness – were observed for items assessing sleepiness during conversation and while eating. Sleepiness in these situations may be interpreted as an indicator of more dysfunctional, potentially neurologically determined forms of somnolence. Such sleepiness reflects a breakdown in the inhibition of wakefulness even in the presence of strong cognitive or emotional stimuli, a phenomenon often characteristic of neurobiologically based disorders such as narcolepsy or idiopathic hypersomnia (8). On this basis, it may be inferred that the ESS-CHAD-SI has latent potential to differentiate between “normal” and “pathological” sleepiness, particularly given that both EFA and CFA suggested a better fit for the two-factor solution. The second factor was primarily defined by items “while eating” and “while talking,” which indicate more clinically concerning aspects of somnolence that are also less common. This factor could thus be labelled concerning sleepiness. The remaining six items loaded on the first factor, which included situations such as reading, watching television or video content, riding in a vehicle, taking an afternoon nap, and after lunch. These represent classic low-stimulation contexts in which sleep deficits are most likely to manifest (8, 34). This factor could thus be termed passive sleepiness. It may be suggested that higher scores on the first factor may reflect inappropriate bedtimes, poor sleep hygiene, or irregular sleep patterns. These causes are modifiable through public health interventions, particularly behavioural strategies aimed at reducing screen time and blue light exposure (e.g., television, computers, mobile phones) and promoting better sleep practices (35,36,37). Conversely, higher scores on the second factor may indicate pathological sleep patterns warranting further diagnostic evaluation, particularly in the context of a history of sleep disorders, sudden daytime sleep episodes, or nocturnal awakenings with disorientation. In such cases, both diagnosing a sleep disorder and implementing pharmacological treatment may be necessary (38).

It can be further argued that the ESS-CHAD-SI is appropriate for rapid screening in schools or counselling centres, where separate assessment of the two dimensions (passive sleepiness and concerning sleepiness) could be particularly beneficial. Children and adolescents with high scores on the second factor but low scores on the first may otherwise remain undetected by general screening scales.

Finally, the ESS-CHAD proved to be reliable, with Cronbach's α coefficients indicating moderate to high internal consistency (31). The results obtained in a sample of American adolescents showed similarly high reliability (13). The slightly higher reliability reported in the original study may partly reflect the fact that the questionnaire was originally developed in English, which can facilitate more precise item interpretation in English-speaking samples. Nevertheless, the reliability coefficients observed in the Slovenian sample were high and fell well within the acceptable range for both research and screening purposes.

In line with the identified two-factor structure, internal consistency estimates calculated separately for each factor indicated acceptable reliability for the passive sleepiness factor and borderline but adequate reliability for the concerning sleepiness factor, supporting the use of both subscales for screening and research purposes.

Although both exploratory and confirmatory factor analyses were conducted on the same dataset, the results should be interpreted cautiously. In this context, the CFA should not be viewed as a strict confirmation of the factor structure, but rather as an additional model-testing step that complements the exploratory findings. Consequently, the two-factor structure identified in the present study should be regarded as a hypothetical model that requires independent confirmation in future samples.

Some potential limitations of the study should be acknowledged. First, although the translation process was conducted according to established guidelines, other methodological approaches, such as involving multiple independent translators or employing a committee-based consensus translation, could have been used and may represent a potential limitation of the current study. However, this limitation is unlikely to have materially affected the results, as the translation and back-translation were performed independently and in a blinded manner by experts with complementary backgrounds, followed by a reconciliation process ensuring semantic, idiomatic, and conceptual equivalence. Although eleven experts from diverse disciplines reviewed the ESS-CHAD-SI, the psychiatry field was not represented. Nevertheless, the expert panel included clinicians and researchers with substantial experience in sleep medicine, neurology, paediatrics, psychology, and public health, which likely mitigated the impact of this omission on the overall content validity assessment. Another limitation is the absence of a linguist or language expert in Slovenian grammar, who could have ensured professional proofreading of the questionnaires. However, the questionnaire underwent cognitive interviewing with adolescents and iterative revisions by native Slovenian speakers with advanced proficiency in English, which helped identify and resolve potential linguistic ambiguities and ensured clarity and comprehensibility of the final version. Furthermore, test-retest reliability was not assessed, as sleepiness and sleep patterns may vary considerably across days; consequently, repeated administration within a short time frame could capture changes in the construct rather than measurement instability. This decision is consistent with previous validation studies of sleep-related self-report instruments and should be interpreted in this methodological context (39). A further limitation is that a separate pilot study on a smaller sample did not precede the psychometric validation. This decision was influenced by contextual constraints related to participant recruitment and data protection in Slovenia, as a sequential design could have adversely affected participation rates and the feasibility of the nationwide study. A methodological limitation of the present study is that the exploratory and confirmatory factor analyses were performed on the same dataset. As a result, the factor structure should be interpreted in an exploratory manner, and its generalizability remains to be established in independent samples. In addition, an orthogonal rotation was applied for reasons of comparability with previous validation studies, although an oblique rotation may have been theoretically justified and could be explored in future research.

While the dataset (40) provides comprehensive coverage of the variables of interest, limitations in sampling density suggest caution in generalising these results beyond the present study population.

Future studies should examine the generalizability of the proposed two-factor structure using independent samples and confirmatory approaches. In addition, alternative factor-retention criteria, such as parallel analysis and scree plots, should be applied to further refine the dimensional structure of the ESS-CHAD-SI.

On the other hand, this study has a notable strength. The two-factor model confirmed that the instrument is not only psychometrically sound, reliable, and construct-valid, but also clinically adaptable for specific applications in healthcare, education, and public health. Owing to these properties, as well as its brevity, the ESS-CHAD-SI can be recommended for screening purposes in paediatric outpatient clinics and schools to identify both passive and concerning excessive daytime sleepiness, for clinical evaluation of EDS (particularly where neurological disorders or sleep disorders are suspected), but the clinical or screening applications should be approached with caution. Our findings strongly support the use of the ESS-CHAD-SI for research and epidemiological purposes.

We suggest that future research should examine the convergent validity of the ESS-CHAD-SI by evaluating its associations with other established measures of daytime sleepiness, particularly the Paediatric Daytime Sleepiness Scale (PDSS). Such analyses would help determine the extent to which the ESS-CHAD-SI aligns with existing instruments and further strengthen its validity in adolescent populations. We also suggest that EDS be examined in the Slovenian adolescent population, as its prevalence is not yet known.

## 5 CONCLUSIONS

In sum, the ESS-CHAD-SI is a psychometrically sound, reliable instrument that captures both public-health-relevant and clinically concerning facets of excessive daytime sleepiness in adolescents. Its brevity and demonstrated validity make it well-suited for integration into routine school health assessments, primary care, and research on sleep health disparities and the effectiveness of interventions. Given the pervasive impact of insufficient and misaligned sleep on cognitive, emotional, and physical development in youth, the availability of a validated Slovenian tool represents a substantive advance for monitoring and improving adolescent sleep health.
